# Intratracheal Poly(I:C) Exposure Accelerates the Immunological Disorder of Salivary Glands in Sjogren's-Like NOD/ShiLtJ Mice

**DOI:** 10.3389/fmed.2021.645816

**Published:** 2021-04-13

**Authors:** Peng Hu, Bingxia Ming, Xuefen Wu, Shaozhe Cai, Jungen Tang, Yuanji Dong, Tianshu Zhou, Zheng Tan, Jixin Zhong, Fang Zheng, Lingli Dong

**Affiliations:** ^1^Department of Rheumatology and Immunology, Tongji Hospital, Tongji Medical College, Huazhong University of Science and Technology, Wuhan, China; ^2^Department of Immunology, School of Basic Medicine, Tongji Medical College, Huazhong University of Science and Technology, Wuhan, China; ^3^Key Laboratory of Organ Transplantation, Ministry of Education, Chinese Academy of Medical Sciences, Wuhan, China; ^4^NHC Key Laboratory of Organ Transplantation, Chinese Academy of Medical Sciences, Wuhan, China; ^5^Key Laboratory of Organ Transplantation, Chinese Academy of Medical Sciences, Wuhan, China

**Keywords:** Sjogren's syndrome, poly(I:C), salivary gland, immune response, IL-33

## Abstract

Evidences have suggested that Sjogren's syndrome (SS) is associated with viral infection. The aim of this study was to investigate the involvement of respiratory viral poly(I:C) in the pathogenesis of SS and potential mechanisms using a SS-like NOD/ShiLtJ (NOD) mouse model. 5-week female NOD mice were intratracheally administered poly(I:C) every other day for 5 times to mimic viral infection. Pilocarpine induced saliva secretion was determined every 8 days. Submandibular glands (SMG) and lungs were harvested for the detection of pathological changes. We found that intratracheal administration of poly(I:C) significantly advanced and enhanced the reduction of saliva flow rate in NOD mice. Furthermore, poly(I:C) treatment aggravated the histopathological lesions and inflammatory cells infiltration in SMG. Accompanied by elevated expression of IFN cytokines and IL-33, Th1 activation was enhanced in SMG of poly(I:C)-treated NOD mice, but Th17 cells activation was unchanged among the groups. In addition, intratracheal poly(I:C) exposure promoted the expression of IL-33 and increased T cells proportion in the lung, which were consistent with the change in SMG. Therefore, intratracheal poly(I:C) exposure aggravated the immunological and function disorder of SMG in NOD mice.

## Introduction

Sjogren's syndrome (SS) is one of the most common rheumatic diseases characterized by chronic inflammation of the exocrine glands, especially salivary and lacrimal glands. Lymphocytic infiltration in the salivary glands usually leads to defective glandular function (1, 2). The prevalence of primary SS is 0.29 to 0.77% in Chinese population (3). Systemic manifestations involving the lung, kidneys, skin and blood systems (4), and the increased risk of B-cell lymphoma (5) are the main causes of poor prognosis and death.

In susceptible individuals, environmental triggers activate the innate immune system (mainly type I interferon (IFN) signature) representing the first stage of SS pathogenesis (6). The stimulus for the activation of type I IFN system in the salivary glands of SS has long been researched (7). Epstein-Barr virus (EBV) encoded small RNA combined with La/SSB from apoptotic salivary gland epithelial cell led to the type I IFN expression via the endosomal RNA sensor TLR3 (5, 8). Numerous independent studies have tested the infections of hepatitis C virus (9), retroviruses and respiratory tract virus [Coxsackie A virus (10, 11), H1N1 vaccination (12), split-virion influenza viral antigens (13)] in patients with SS. Animal research showed an upregulated expression of TLR3 and type I IFN in submandibular glands and SS-like sialadenitis in NZB/WF1 mice after intraperitoneal administration of poly(I:C) (14, 15). Zhou et al. found that intraperitoneal poly(I:C) treatment resulted in pathology of SS-like dacryoadenitis in non-autoimmune-prone C57BL/6 mice (16). These data suggest that SS is associated with viral infection. However, it is unclear the role and potential mechanisms of respiratory tract virus infection in the alteration of glandular function.

Polyinosinic: polycytidylic acid [poly(I:C)], a synthetic double stranded RNA, sensed by TLR3, has been widely used to mimic virus infection (17). Poly(I:C) stimulation could induce the release of IL-33 in other conditions (18, 19), which has been reported to be increased and acts with IL-12 and IL-23 to favor the secretion of IFN-γ in SS (20). The NOD/ShiLtJ (NOD) mouse model, spontaneously developing SS-like symptoms, is widely used for investigating SS (21). The earliest incidence of sialadenitis in submandibular glands (SMG) of NOD mice occurs at 6 to 7 weeks, while elevated blood glucose mainly occurs after 15 weeks (22). In this study, we found that intratracheal stimulation of poly(I:C) aggravated salivary gland dysfunction in spontaneous SS-like NOD mice. IFN cytokines and T cell chemokines were upregulated, along with an increased expression of IL-33 in salivary gland. Interestingly, poly(I:C) exposure also increased the IL-33 expression and T cells proportion in the lung. These data suggest that intratracheal poly(I:C) exposure aggravated the immunological and function disorder of SMG to promote SS-like progression.

## Materials and Methods

### Mice

This study was performed in compliance with the guidelines of Institutional Animal Care and Use Committee (IACUC) at Tongji Hospital (Wuhan, China). Five-week-old female NOD/ShiLtj (NOD) mice were purchased from Hua Fu Kang Bioscience company (Beijing, China) and allowed to maintain in the specific pathogen-free facility. The anesthetized NOD mice were in a hypsokinesis of head and vertical position, the tongue of mice was gently fixed to expose the root, 20 μl sterile PBS or poly (I:C) (1 mg/ml) was inhaled into the lung through the airway with a micropipette auxiliary. Poly(I:C) (InvivoGen Corp, San Diego, CA, USA) was administered each time with 20 μg on day 0, 2, 4, 6, 8. The sterile PBS-treated mice and untreated mice were used as controls. Pilocarpine (Abcam Corp, Cambridge, UK) (1 mg/ml) induced saliva volume was determined every 8 days on day 0, 9, 16, 24 and 32. SMG and lungs were harvested on the 52th day for further detection.

### Saliva Flow Rate Measurement

Saliva flow rate was detected as previously described (23). Briefly, NOD mice were anesthetized and then intraperitoneally injected with pilocarpine (Sigma-Aldrich) of 5 mg/kg body weight. Total saliva was collected from the oral cavity for 10 min after pilocarpine stimulation, and then quantified as the volume. The saliva flow rate was presented as saliva volume to body weight for each individual.

### Gene Expression Analysis

Quantitative real-time PCR was used to determine the gene expression levels in SMG and lung. Total RNA was obtained from tissues and then reverse transcribed to cDNA by RevertAid First Strand cDNA Synthesis Kit (Thermo Corp, Waltham, MA, USA) according to the manufacturer's instruction. The expression *of IFN-*α*, IFN-*β*, IFN-*γ*, IFN-*λ*, TNF-*α*, IL-33, IL-17A, CXCL9, CXCL10, CXCL11, CXCL13 and* β*-actin* (Primer from TsingKe Corp, Wuhan, China) (Table 1) was determined by SYBRGreen quantitative real-time PCR (TOYOBO Corp, Osaka-Shi, Japan). β*-actin* housekeeping gene was used as a control. The relative gene expression was calculated by using the 2^∧^ (−ΔΔCt) method.

**Table 1:** The primer sequences of the genes.

**Gene**	**Primer sequences**	**Tm values**
IL-33	Forward TTCCAACTCCAAGATTTCCCC Reverse CAGAACGGAGTCTCATGCAG	57.83 58.36
IFN-α	Forward AGCGCCGTCTAAGATTCATGT Reverse CGTCGTGGCAATTCTAGTGG	59.86 59.00
IFN-β	Forward TCAGAATGAGTGGTGGTTGC Reverse GACCTTTCAAATGCAGTAGATTCA	58.10 57.50
IFN-γ	Forward CTTTGGACCCTCTGACTTGAG Reverse TCAATGACTGTGCCGTGG	57.95 57.62
IFN-λ	Forward AGCTGCAGGCCTTCAAAAAG Reverse TGGGAGTGAATGTGGCTCAG	59.32 59.67
CXCL9	Forward AGTCCGCTGTTCTTTTCCTC Reverse TGAGGTCTTTGAGGGATTTGTAG	57.83 57.83
CXCL10	Forward TCAGCACCATGAACCCAAG Reverse CTATGGCCCTCATTCTCACTG	57.66 57.88
CXCL11	Forward ATGGCAGAGATCGAGAAAGC Reverse TGCATTATGAGGCGAGCTTG	57.76 58.70
CXCL13	Forward AGATCGGATTCAAGTTACGCC Reverse ACAGACTTTTGCTTTGGACATG	58.18 57.69
IL-17A	Forward AGGCCCTCAGACTACCTCAACC Reverse GCCTCTGAATCCACATTCCTT	63.16 57.99
TNF-α	Forward CATCTTCTCAAAATTCGAGTGACAA Reverse TGGGAGTAGACAAGGTACAACCC	58.11 61.33
β-actin	Forward GGTCAGAAGGACTCCTATGTGG Reverse TGTCGTCCCAGTTGGTAACA	59.57 58.88

### Histological Analysis and Immunohistochemistry

SMG and lung tissues were collected in 4% paraformaldehyde and embedded in paraffin. Paraffin-embedded tissue sections (5 μm) were stained with hematoxylin and eosin (H&E) staining, an aggregation of inflammatory cells >50 was considered as a foci in SMG. The cross-sectional characteristics of SMG, quantified by focus scores and the proportion of inflammatory cells aggregation area to total SMG area, were evaluated. The severity of tissue damage was evaluated by a scoring system based on the degree of lymphoepithelial lesions (LELs) (24). Standard immunohistochemical (IHC) staining was conducted to evaluate the expression of CD3 in the lung tissues with anti-CD3 monoclonal antibody (1:200 dilution, Servicebio Corp, Wuhan, China), and IL-33 in SMG and lung tissues with anti-IL-33 polyclonal antibody (1:800 dilution, R&D systems, Minneapolis, MN, USA). The sections were analyzed by ImageJ software. Three slides from each tissue were screened by two pathologists who were blind to the group inflammation.

### Statistical Analysis

All data were presented as mean ± standard error of the mean (SEM). One-way ANOVA analysis was used for multi-group test after passing the normality tests (Shapiro-Wilk tests). SPSS software (version 19.0) was used for statistic analysis, and a value of *p* < 0.05 was considered as statistically significant.

## Results

### Poly(I:C) Intratracheal Administration Enhanced the Reduction of Saliva Flow Rate in NOD Mice

**Figure 1:**
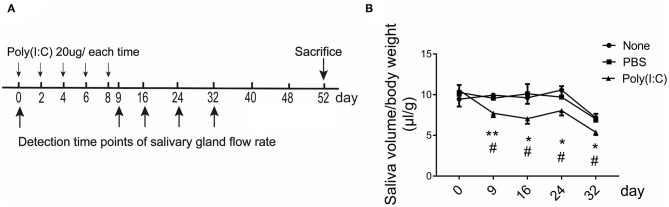
The saliva flow rate in NOD mice intratracheally treated with poly(I:C). **(A)** Administration regimen for the induction of poly(I:C)-treated mice. **(B)** Pilocarpine-induced saliva volume was determined every 8 days. The number of the three groups were 6, 6, and 8, respectively. Data were presented as mean ± SEM, *poly(I:C) vs. none, *p < 0.05, **p < 0.01; ^#^poly(I:C) vs. PBS, ^#^*p* < 0.05.

To examine whether intratracheally administered poly(I:C) has an effect on salivary glands function, NOD mice were repeatedly administered poly(I:C) intratracheally, and pilocarpine-induced saliva volume was determined at day 0 (5-week mice) and following every 8 days. The mice were sacrificed at day 52 (12-week mice), as SMG infiltration was obvious and without occurrence of diabetes at 12 weeks of age (Figure 1A and Supplementary Figure 1). As shown in Figure 1B, poly(I:C)-treated mice had a significant reduction in the saliva volume from the early stage until 32 days compared with the untreated and PBS-treated mice, the saliva flow rates of the control groups were normal until 24 days. There was no weight loss and mortality appeared among the groups. The above data suggest that poly(I:C) treatment leads to an advance of the onset of SS-like symptom.

### Treatment With Poly(I:C) Aggravated the Histopathological Lesions in Salivary Gland

**Figure 2:**
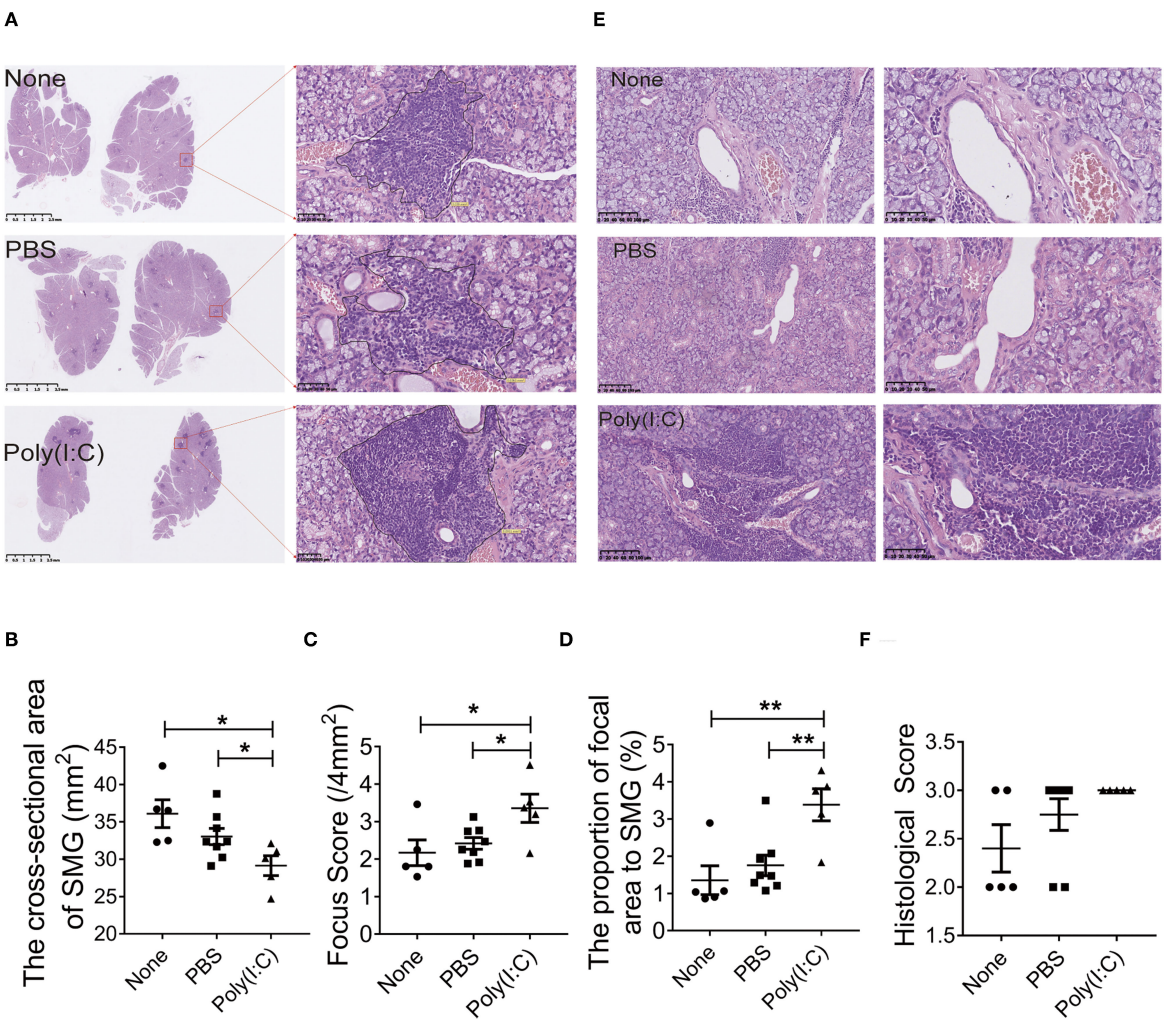
Treatment with poly(I:C) aggravated the histopathological lesions of salivary gland. **(A)** Infiltrated lymphocyte foci in the acinar tissue of salivary gland in different groups (HE, ×10, ×400) (*n* = 5–8 per group). **(B)** The analysis of the cross-sectional area of SMG in different groups. **(C)** The analysis of the focus score in different groups. **(D)** The analysis of the proportion of inflammatory cells area to the total salivary gland area in different groups. **(E)** Histological destruction of glandular duct (HE, ×100, ×200). **(F)** Histological destruction was assessed by a classification system with the proportion of the hyperplastic epithelium per square millimeter (mm^2^). Data were presented as mean ± SEM, *p < 0.05, **p < 0.01.

To determine the effect of poly(I:C) administration on the histopathological lesions of salivary gland in NOD mice, sections of salivary gland harvested at day 52 were stained with H&E staining. As shown in Figures 2A,B, the cross-sectional area of salivary gland in poly(I:C) treated group was smaller than that in PBS-treated or untreated group, indicating that the volume of gland was reduced after poly(I:C) administration. There were more inflammatory cells foci in salivary gland per 4 mm^2^ (focus score) in poly(I:C)-treated group (Figure 2C). The proportion of inflammatory cells aggregation area in total salivary gland cross-sectional area was increased after poly(I:C) administration (Figure 2D). Furthermore, we analyzed the histopathological change of another two sections every 10 μm interval in each salivary gland, and similar results were observed (data not shown). Poly(I:C) treatment increased CD3-positive T cells accumulation and unchanged the CD20-positive B cells accumulation in the salivary glands (Supplementary Figure 2). The lymphoepithelial lesions (LELs), evaluated by the proportion of the hyperplasia of epithelium resulted from infiltrated lymphocytes, was more serious in intratracheal poly(I:C)-treated group (Figures 2E,F). These data suggest that intratracheal poly(I:C) administration accelerated the histopathological changes of salivary glands in NOD mice.

### Poly(I:C) Treatment Increased IFN Cytokines and T Cells Chemokines Levels in Salivary Gland

**Figure 3:**
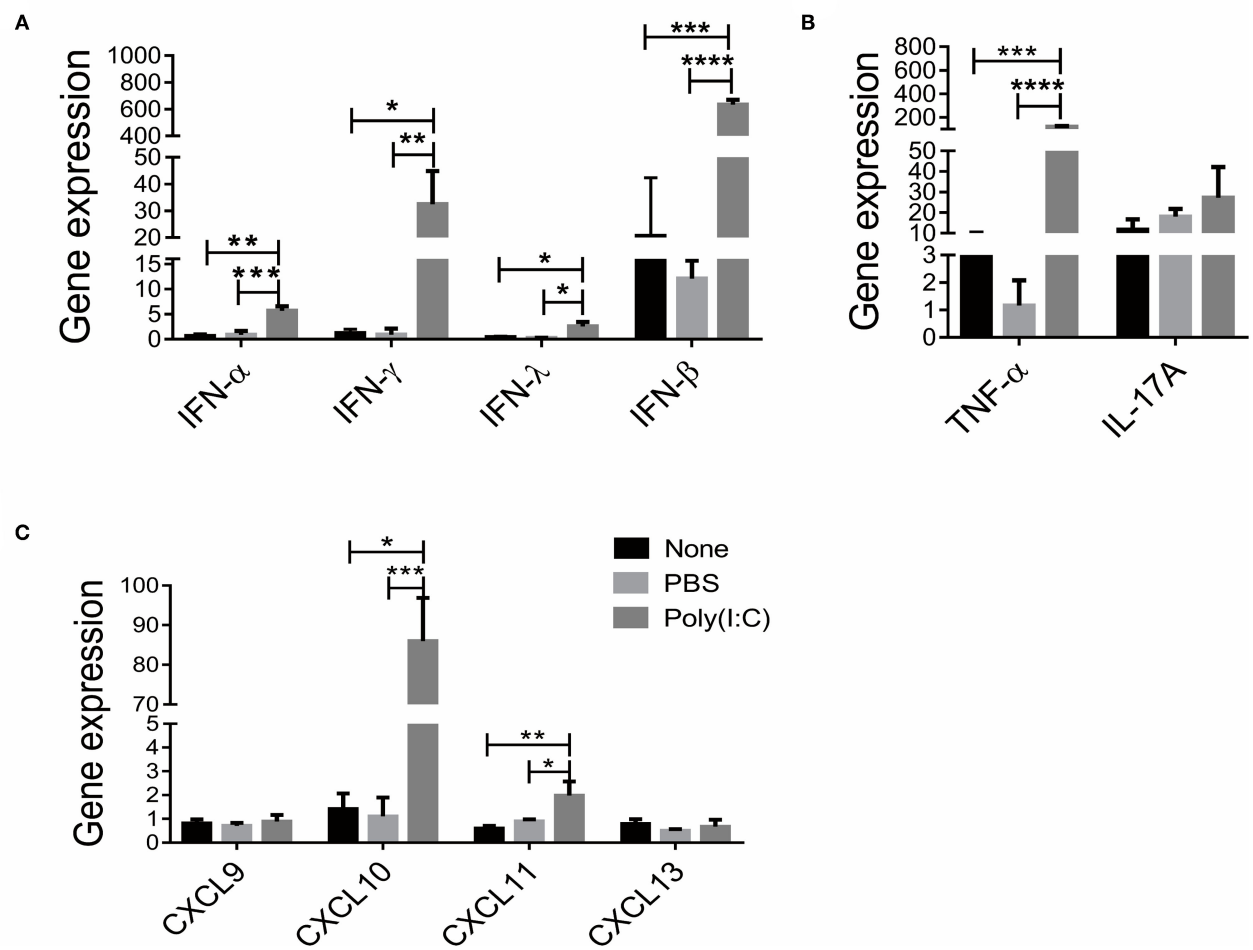
Poly(I:C) treatment increased IFN cytokines and T cell chemokines levels in salivary gland. **(A)** The mRNA levels of IFN (IFN-α, IFN-β, IFN-γ, IFN-λ) were determined by real-time PCR (*n* = 5, 5, 4). **(B)** The mRNA levels of T cell associated cytokines were determined by real-time PCR, TNF-α (Th1 cytokine) and IL-17A (Th17 cytokine). **(C)** The mRNA levels of CXCL9, CXCL10, CXCL11 and CXCL13 in salivary gland. Data were presented as mean ± SEM, *p < 0.05, **p < 0.01, ***p < 0.001, and ****p < 0.0001.

Previous studies have reported that IFN, Th1 and Th17 signaling participated in the development of SS (25–27). We then investigated the immune status in salivary gland after poly(I:C) administration. As shown in Figure 3A, significantly upregulated expressions of *IFN-*α*, IFN-*β*, IFN-*γ and *IFN-*λ, particularly *IFN-*β (72.3-fold) were observed in respiratory tract poly(I:C)-treated group. Furthermore, the expression of *TNF-*α, a Th1 cell associated cytokine, was increased compared with the control groups, though the expression of *IL-17A*, a Th17 cell associated cytokine, was unchanged after poly(I:C) treatment (Figure 3B). We further detected the expression of T cell chemotactic factors *CXCL9, CXCL10, CXCL11* and B cells chemotactic factor CXCL13. As shown in Figure 3C, *CXCL10* and *CXCL11* expression were elevated in poly(I:C)-treated group, while *CXCL9* and *CXCL13* expression were not different from each group. The level of serum autoantibody ANA was comparable among the groups (Supplementary Figure 3). These data suggest that intratracheal poly(I:C) administration affected IFN signal and Th1 cell accumulation in salivary gland, which is consistent with virus infection-induced immune response.

### Poly(I:C) Intratracheal Stimulation Upregulated the Expression of IL-33 in Salivary Gland

**Figure 4:**
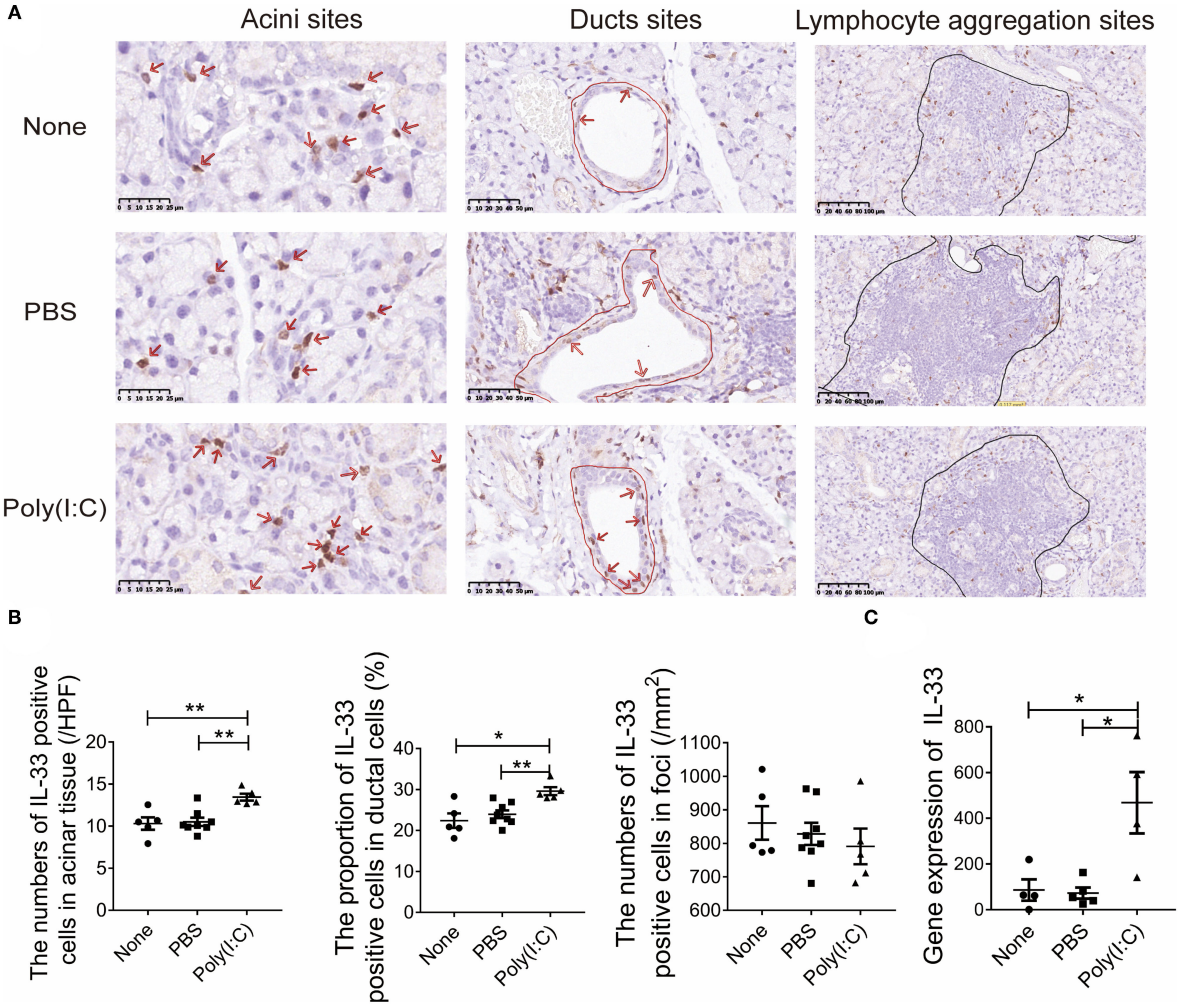
Poly(I:C) intratracheal stimulation upregulated the expression of IL-33 in salivary gland. **(A)** Immunohistochemical staining for IL-33 expression in salivary gland (×400, ×400, ×200) (*n* = 5–8 per group). **(B)** The number of IL-33 positive cells per high power field (HPF) in acini sites, the proportion of IL-33 positive cells to total ductal cells in ducts sites, the number of IL-33 positive counts in lymphocyte aggregation sites per square millimeter (mm^2^). **(C)** The mRNA level of IL-33 in salivary gland (*n* = 5, 5, 4). Data were presented as mean ± SEM, *p < 0.05, **p < 0.01.

Poly(I:C) can induced the expression and release of IL-33, a damage associated molecular patterns (DAMP). Studies have shown that IL-33 is involved in Th1 cell response (28). The expression of IL-33 in salivary gland were detected by immunohistochemistry (Figure 4A). We found that the number of IL-33 positive cells per high power field (HPF) in acini sites was increased after poly(I:C) administration, the proportion of IL-33 positive cells to total ductal cells in ducts sites was also increased, though there was no difference in the lymphocyte aggregation sites among the three groups (Figure 4B). Meanwhile, the mRNA level of IL-33 expression in salivary gland was higher in poly(I:C)-treated mice (Figure 4C). Hence, intratracheal poly(I:C) treatment resulted in an upregulation of IL-33 expression, which might promote the Th1 cell response in salivary gland.

### Poly(I:C) Treatment Increased IL-33 Expression and T Cells Proportion in the Lung

**Figure 5:**
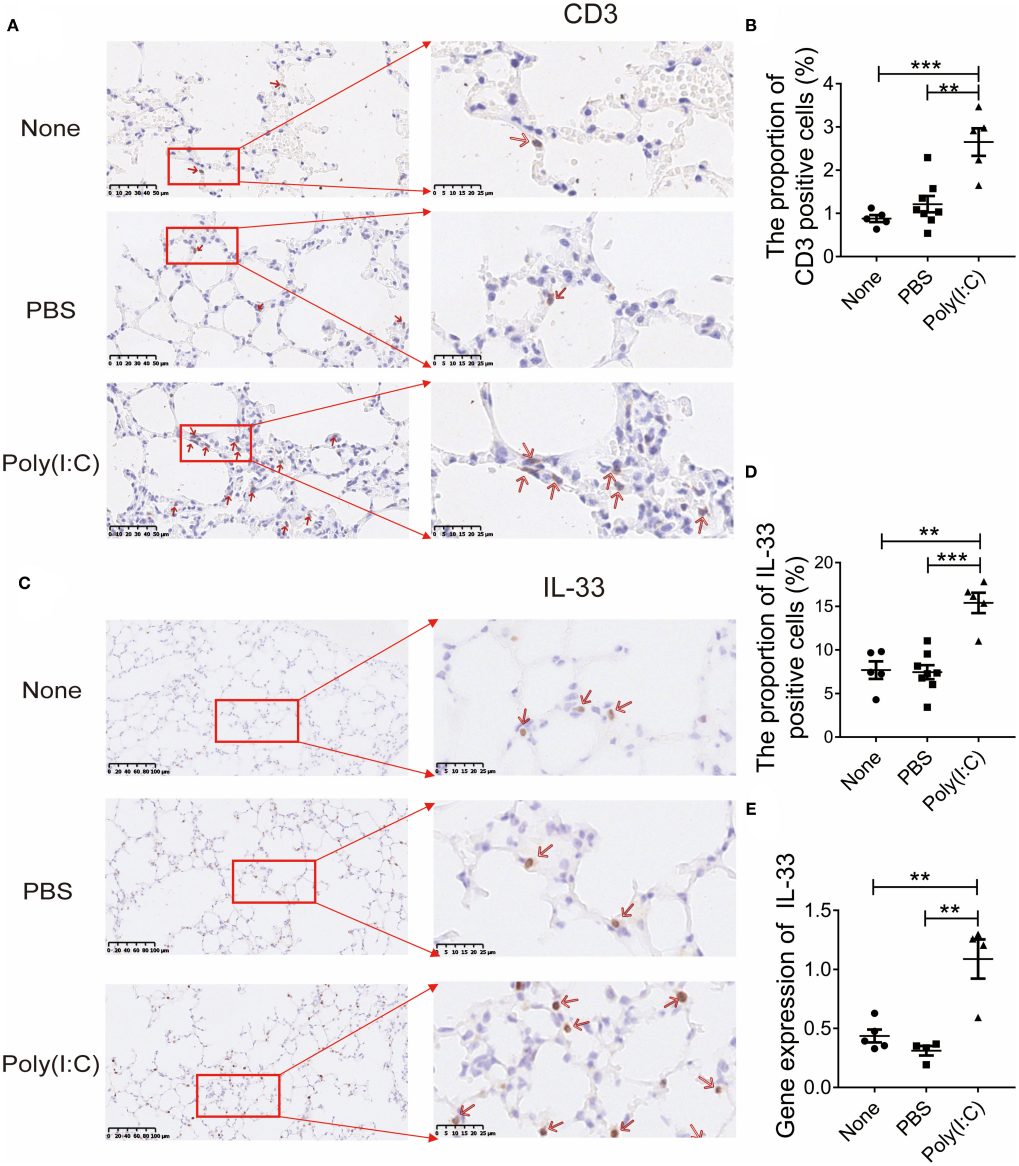
Poly(I:C) treatment increased IL-33 expression and T cells proportion in the lung. **(A)** Immunohistochemical staining for CD3 expression in lung (×400, ×800) (*n* = 5–8 per group). **(B)** The proportion of CD3 positive cells to total cells in lung per HPF. **(C)** Immunohistochemical staining for IL-33 expression in lung (×200, ×800) (*n* = 5–8 per group). **(D)** The proportion of IL-33 positive cells to total cells per HPF. **(E)** The mRNA levels of IL-33 in lung (*n* = 5, 5, 4). Data were presented as mean ± SEM, **p < 0.01 and ***p < 0.001.

To observe the changes of lung which may be associated with salivary gland injury after poly(I:C) stimulation, we further explored the indicator in the lung. The sections of lung were stained with anti-CD3 antibody, the results showed the increased CD3-positive T cells accumulation in interstitium of lung (Figures 5A,B), in addition to aggravated mucus secretion and bronchial thickening compared with control groups (Supplementary Figure 4). We further detected the expression of IL-33 in the lung. The number of pulmonary IL-33 positive cells per HPF was increased obviously (Figures 5C,D). The expression of IL-33 in mRNA level in the lung was higher after poly(I:C) administration (Figure 5E). These data indicate that poly(I:C) stimulation increased IL-33 expression and T cells proportion in the lung, which are similar to the change in salivary gland.

## Discussion

It is generally believed that viral infection may be an important environmental factor in genetically susceptible individuals of SS. Among them, respiratory virus infection including vaccinations and enterovirus may participate in the development of SS (10, 11). Studies have shown that poly(I:C) administration is a well-established model to mimic viral infection in systemic lupus erythematosus (29), type 1 diabetes (30), and arthritis (31) animal model. In this study, we found that repeatedly intratracheally administered poly(I:C) in susceptible NOD mice advanced the onset of sialadenitis, accelerated the histopathological lesions of SMG. Further analysis showed that the IFN signature and Th1 immune response were upregulated in the local of SMG. IL-33, which is participated in viral infection, was increased in SMG. Interestingly, the expression of IL-33 and T cells were also elevated in the lung, which is consistent with the change in SMG. Thus, respiratory tract viral infection might be involved in the etiopathogenesis of SS-like progression.

Lymphocytic infiltration of salivary glands is the hallmark of SS, and saliva volume is used to evaluate the function of salivary gland. In this study, we found that there was a significant reduction of saliva production in poly(I:C)-treated mice until day 32. The pathological lesion and lymphocyte infiltration in salivary glands in poly(I:C)-treated mice remained at the end of the study (52th day). We can see this phenomenon in other studies. Intraperitoneal administration of poly(I:C) in NZB/WF1 mice caused the reduction of saliva compared with untreated group (15), accompanied with more severe lymphocytic infiltration (14). Freund's incomplete adjuvant (IFA) stimulation resulted in the mild sialadenitis while significant glandular hypofunction (32). Even the exocrine gland dysfunction is considered as a process independent from inflammation in the pathogenesis of SS (33). These results indicate that the dysfunction of salivary glands presented with reduction of saliva results from mainly lymphocytic infiltration and other factors.

T lymphocytes play an important role in glandular damage and disease progression in SS (34), Activated CD4^+^T cells can mediate the local inflammatory responses and activate B cells to promote the production of plasma cells and autoantibodies (35). In the present study, the effects of intratracheal poly(I:C) administration on Th1-related chemokines and inflammatory cytokines production within the SMG were investigated. Poly(I:C) stimulation caused significant upregulation in the expression of Th1-related chemokines CXCL10 and CXCL11 genes that influence the inflammatory cell infiltration within the SMG. The expression levels of Th1-related cytokines TNF-α was also upregulated. The abnormality of IFN signature has been reported in the blood and salivary glands of patients with Sjogren syndrome (36). Poly(I:C) stimulation increased the expression levels of IFN genes, especially IFN-β, in SMG. The activation of IFN and Th1 response occurred in the condition of viral infection, which might explain the accelerated progression of salivary gland dysfunction in NOD mice after poly(I:C) stimulation.

Sjogren's syndrome is characterized by production of autoantibodies. We found that poly(I:C) stimulation unchanged the production of ANA compared with control groups in NOD mice. Accordingly, the B cell chemokine CXCL13 expression and CD20-positive B cells were unchanged in salivary glands after respiratory tract poly(I:C) stimulation. These factors may be associated with the unchanged production of ANA in poly(I:C)-treated NOD mice.

In the present study, intratracheal poly(I:C) stimulation increased IL-33 expression and T cells infiltration in the lung, which was similar to the changes observed in salivary glands. Therefore, we speculate that there was a link between lung and salivary glands, which resulted in the dysfunction of salivary glands in poly(I:C)-exposed NOD mice. IL-33, a DAMP, can be induced and released from epithelial or endothelial cells in respiratory virus infection (37, 38). Other studies have found that IL-33 promotes the differentiation and function of Th1 type cells, including the upregulated expression of IFN-γ, IL-18, t-bet and CXCR3 in influenza virus infection condition (37, 39). We found that IL-33 detected by IHC and real time-PCR both showed increased expression in lungs and salivary glands after poly(I:C) administration. Salivary glands and respiratory tract tissues belong to the mucosal immune system (40, 41). Thus, we speculate that the cytokine, such as IL-33, or activated T cells from lungs to salivary glands may result in the immune dysfunction and glandular destruction, which needs to be further investigated.

In conclusion, our results showed that intratracheal exposure of poly(I:C) results in a significant loss of glandular function, severe histopathological lesions and immune imbalance in salivary glands, indicating that intratracheal poly(I:C) exposure aggravated the immunological and function disorder of SMG to promote SS-like progression.

## Data Availability Statement

The raw data supporting the conclusions of this article will be made available by the authors, without undue reservation.

## Ethics Statement

The animal study was reviewed and approved by the guidelines of Tongji Hospital Animal Care and Use Committee.

## Author Contributions

LD and FZ designed the study. PH and BM performed the experiments, analyzed the data, and wrote the paper. XW, SC, JT, YD, and TZ helped for bleeding the mice and samples acquired. ZT and JZ contributed to the interpretation of the data. All authors read and approved the final manuscript.

## Conflict of Interest

The authors declare that the research was conducted in the absence of any commercial or financial relationships that could be construed as a potential conflict of interest.
