# Supplementary figures and images for: Intratracheal Poly(I:C) Exposure Accelerates the Immunological Disorder of Salivary Glands in Sjogren's-Like NOD/ShiLtJ Mice
Source: Front Med (Lausanne). 2021 Apr 13;8:645816. doi: 10.3389/fmed.2021.645816 (PMC8076562; doi:10.3389/fmed.2021.645816)

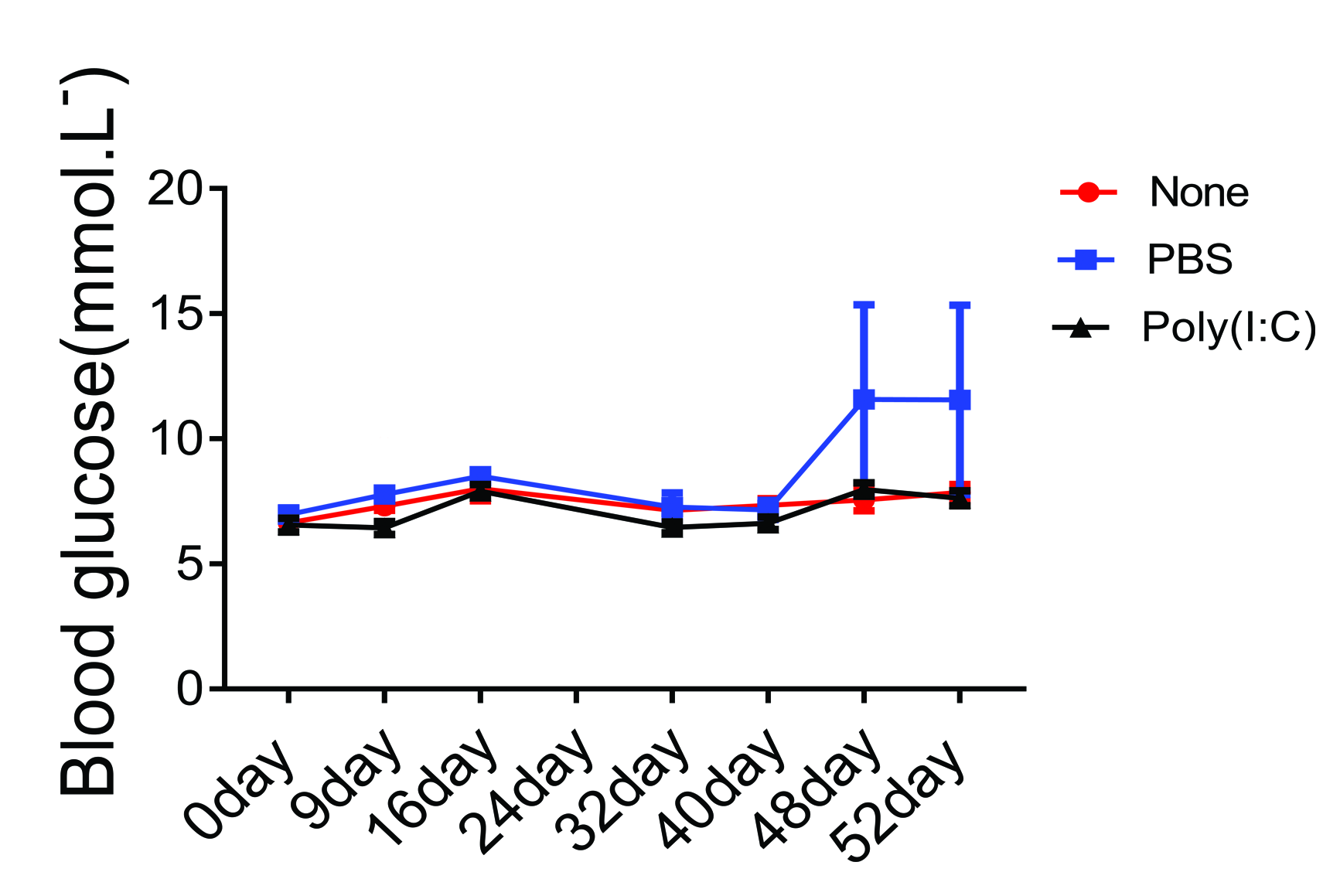

Supplement: Supplementary file 1 [file Image_1.TIF]

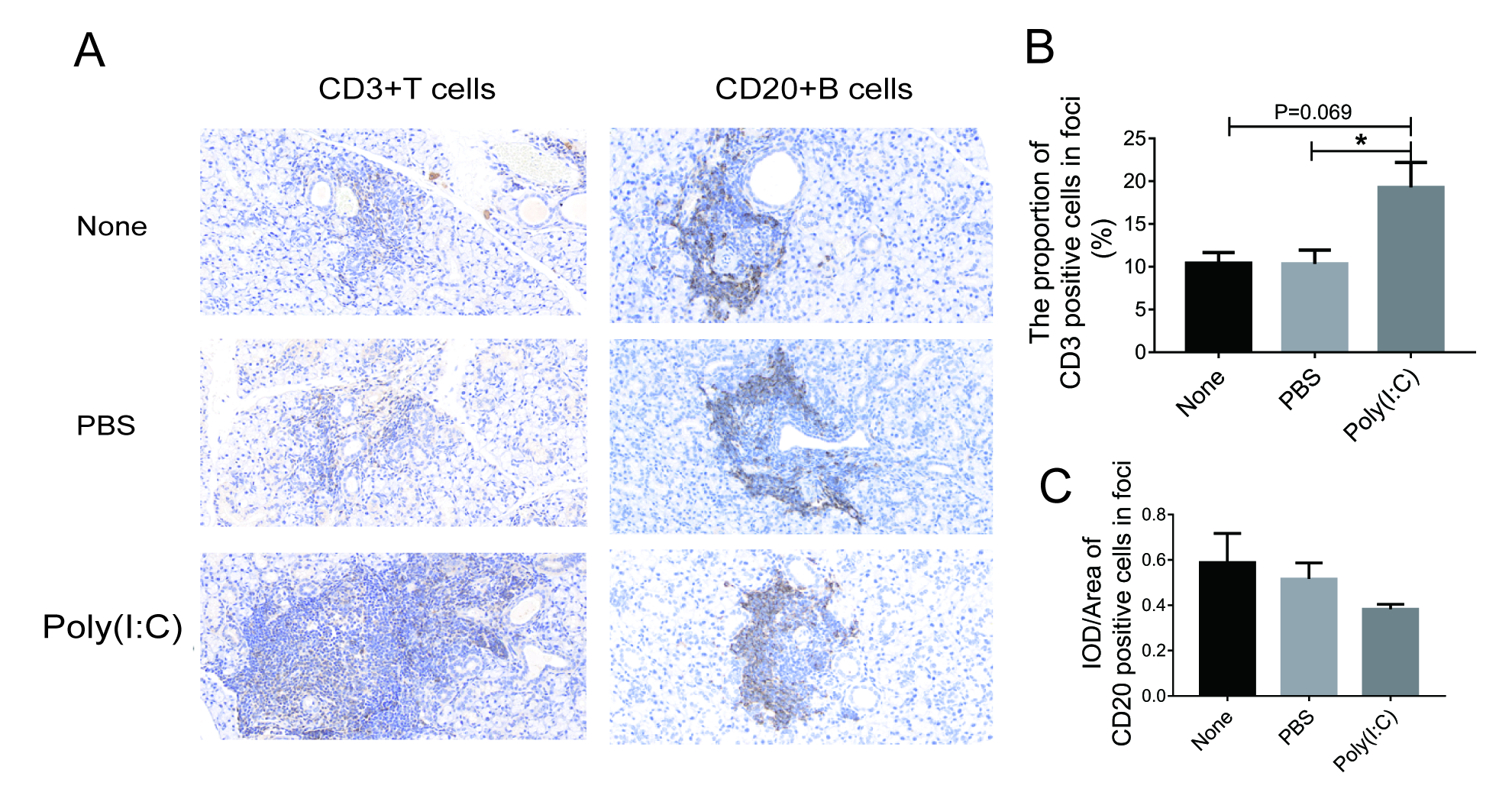

Supplement: Supplementary file 2 [file Image_2.TIF]

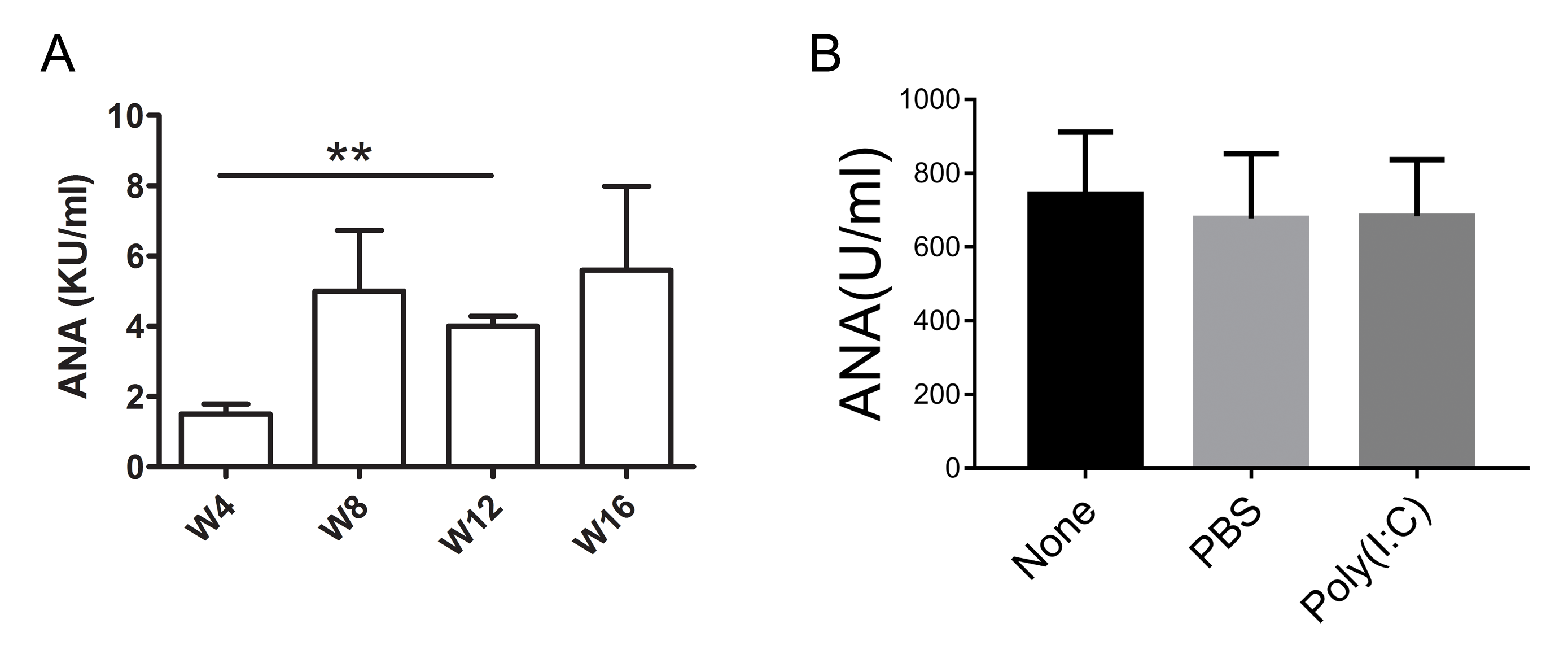

Supplement: Supplementary file 3 [file Image_3.TIF]

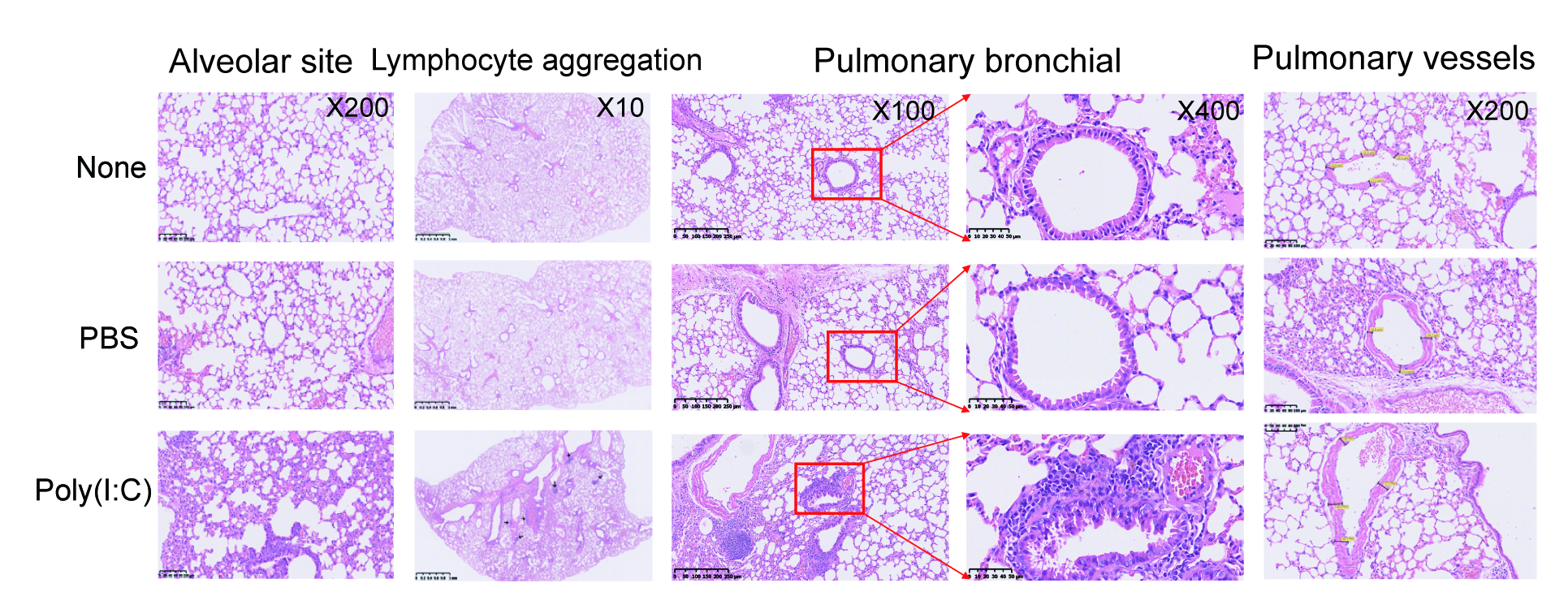

Supplement: Supplementary file 4 [file Image_4.TIF]
